# Predictors for response to electronic patient-reported outcomes in routine care in patients with rheumatoid arthritis: a retrospective cohort study

**DOI:** 10.1007/s00296-023-05278-6

**Published:** 2023-01-30

**Authors:** Jimmy Wiegel, Bart F. Seppen, Michael T. Nurmohamed, Marieke M. ter Wee, Wouter H. Bos

**Affiliations:** 1 Amsterdam Rheumatology & Immunology Center, Reade, Admiraal Helfrichstraat 1, 1056 AA Amsterdam, The Netherlands; 2 Amsterdam Rheumatology & Immunology Center, Amsterdam UMC Location VUmc, Amsterdam, The Netherlands; 3 Amsterdam UMC Location Vrije Universiteit, Department of Epidemiology & Data Science, Boelelaan 1117, Amsterdam, The Netherlands; 4 Amsterdam UMC Location Vrije Universiteit, Department of Rheumatology and Immunology, Boelelaan 1117, Amsterdam, The Netherlands; 5 Amsterdam Public Health, Methodology, Societal Participation in Health, Amsterdam, The Netherlands; 6 Amsterdam Institute for Infection and Immunity, Inflammatory Diseases, Amsterdam, The Netherlands

**Keywords:** Electronic patient reported outcomes, ePROs, Adherence, Rheumatoid arthritis, eHealth, Telehealth

## Abstract

Routine collection of electronic patient-reported outcomes (ePROs) can improve clinical care. However, a low response rate may counteract the benefits. To optimize adoption, the aim of this study was to investigate which patient factors and/or timing of the invitation predicted response to ePROs sent prior to consultations in patients with rheumatoid arthritis. We performed a retrospective database study with clinical data collected as part of usual care from the electronic medical records at Reade Amsterdam. The dataset comprised the email invitations to complete the ePRO sent prior to consultation. Multiple patient factors and factors defining the timing of the invitation were investigated if they predicted response to the ePRO through a multivariable logistic generalized estimating equation analysis. In total, 17.070 ePRO invitations were sent to 3194 patients (mean age 60 (SD 14), 74% female), of which 40% was completed. Patients between 55 and 73 years (OR 1.39, 95%CI 1.09–1.77) and with higher social economic status (SES) (OR 1.51, 95%CI 1.22–1.88) had significantly higher odds for completing the ePRO, while patients living in an urban area had lower odds (OR 0.69, 95% CI 0.62–0.76). In year 4 after implementation, the OR was increased to 3.69 (95% CI 2.91–4.90). The implementation of ePROs in daily clinical practice needs improvement since 40% of the ePROs sent prior to consultations were completed. Patients that had higher odds to report the next ePRO were between the age of 55–73, had a higher socio-economic status, and were residents in a rural area. The adoption of reporting the PRO increased over time, but the timing of the prompt did not predict response. Additional research is needed to understand ePRO completion, especially for patients with lower socio-economic status.

**Supplementary Information:**

The online version contains supplementary material available at 10.1007/s00296-023-05278-6.

## Introduction

Systematic monitoring of patient-reported outcomes (PROs) has the potential to improve the daily clinical practice of patients with rheumatoid arthritis (RA). Trial results showed that PROs can improve patient-provider communication, monitor treatment (side) effects, detect unrecognized problems, improve patient satisfaction and may even reduce the number of clinical consultations [1–3]. Electronic PROs (ePROs) are thought to help the integration of PROs into clinical care by automating routine collection [4, 5]. Furthermore, they can be integrated into electronic medical records, making it easier to use the results during consultations by clinicians.

Despite these promising results in clinical trials, ePROs have not yet found broad application in routine clinical practice, mainly due to a low completion rate by patients [6]. Recent trials showed a decreasing completion of ePROs over time, down to 40% after six months [3, 7, 8]. To date, only a little evidence is available on which patient factors are associated with a lower or higher ePRO response rate. A recent study showed that patients with a lower social economic status (SES) and living in rural areas were important predictors for lower online patient portal usage [9]. For ePRO completion specific, it was found that older age was associated with a higher response rate, while in another study we performed ourselves, we found that women tend to stop sooner with reporting ePROs and completed less ePROs [7, 8]. However, these results were often affected by a selection bias due to an underrepresentation of patients with a lower SES (i.e. patients with lower education or lower eHealth literacy) [[7, 8, 10]. In addition to patient factors, in a recent qualitative study patients mentioned that also the prompt timing to report the ePRO (the time of the day or day of the week on which the ePRO invitation is sent), influences the likelihood that they complete the ePRO [11]. Although prompts are indeed frequently associated with increased eHealth usage, the relation between the timing of the prompt and the completion of ePROs is only scarcely investigated and is unknown [12, 13]. As well as unclarity and little existing knowledge on which patient factors are of importance in the collection of ePROs in routine clinical practice. By identifying which patient groups do not complete their ePRO, we may facilitate targeted improvements to optimize the adoption and, therefore, might overcome possible patient disparities regarding ePRO completion. Furthermore, insight into the preferred timing of the prompt may help to facilitate reporting ePROs.

Therefore, we studied which patient factors and/or timing of the prompt predict response to ePROs sent prior to the consultation in routine clinical care in patients with RA.

## Methods

### Study design

We performed a retrospective database study with data from the electronic medical records between June 2016 and October 2020 at Reade center for Rheumatology and Rehabilitation in Amsterdam, the Netherlands. Since June 2016 each patient is invited by an automated email one week before their consultation at the clinic to complete a Multi-Dimensional Healthcare Assessment Questionnaire (mMDHAQ) via the Reade patient portal [14]. The results are sent in real-time to the medical records which give the rheumatologist insight into the overall wellbeing of the patient, the disease activity, and any remarks the patient would like to discuss before and during consultation. This study was performed using readily available clinical patient records of Reade. All collected data were anonymized. The procedures in this investigation were in accordance with Dutch legislation (the Medical Research Involving Human Subjects Act), ethical standards on human experimentation in the Netherlands and local data protector concerning the privacy of the patients.

### Study outcome

The outcome was defined as ePRO completion for each sent invitation separately. Only when the ePRO was completed, in other words, all questions were answered, the results of the ePRO were sent to the medical record, and the ePRO invitation was coded as “completed”, otherwise it was coded as “uncompleted”.

### Study population

We selected patients diagnosed with RA according to the 10th revision of the International Classification of Diseases (ICD-10) criteria (M06.99 RA unspecified, M06.09 RA without Rheumatoid Factor, or M05.99 seropositive RA). Patients who had no registered email address were excluded since they could not receive the invitation. No other exclusion criteria were used.

### Investigated factors

The investigated factors were based on the results of previous studies. However, since we used pre-existing data, we were limited to those factors that were available in the medical records.

#### Patient factors

The investigated patient factors comprised age, sex, residing distance to Reade, SES and disease duration. Age was categorized according to the corresponding generation: 18–38 years (“millennials”), 39–54 years (“gen X), 55–73 years (“Baby boomers”), and 74 years and older (“silent generation”) [9]. Urban or rural residency was dichotomized to living in Amsterdam (urban) or out of Amsterdam (rural). SES was derived from the publicly available dataset of the Amsterdam municipality as the disposable household income and was therefore only calculated for patients living in Amsterdam [15]. This dataset encompassed the average disposable household income per district of Amsterdam [16]. Patients were classified in their according to districts based on their zip code. The districts were categorized into quartiles to create better interpretable effect sizes, from lowest average income (one) to highest (four). Disease duration was calculated for each separately sent ePRO based on the reported diagnosis date in the medical record. Due to a recent change in EMR, we could only accurately determine the reported diagnosis date up to 4 years, hence we categorized the diagnosis date into < 1 year, 1 year, 2 years, 3 years, 4 years and > 4 years.

#### Timing of the prompt

The year, the day of the week and the period of the day the ePRO were sent were all analyzed as independent factors. Time of the day the ePRO was sent was dichotomized into working hours (9–17 h) and non-working hours.

### Statistical analysis

Measures were given as mean and standard deviation (SD) if normally distributed, otherwise the median and interquartile range (IQR) were calculated. Since we only had information in SES for patients living in Amsterdam, we build two prediction models. Model 1 encompassed all patients including the factor “urban” or “rural” as a marker for residing distance to Reade, but excluding SES. Model 2 encompassed urban patients only and included the SES as a factor. Since patients could have repeated measures over time, we applied a multivariable logistic Generalized Estimating Equation (GEE) with an exchangeable correlation structure. This longitudinal GEE analysis corrects for dependent observations within a person. A time-lag model related the result of a possible predictor to report the ePRO to the following sent ePRO. All factors were selected for the multivariable logistic GEE model since a large number of records and a limited number of factors. A manual backward selection procedure was performed, with a *p*-value of 0.01 as cut-off value. When a continuous variable is selected in the prediction model, the relation between the variable and the outcome is checked for linearity by dividing the continuous variable into quartiles. When the relation is not linear, the quartiles are presented. The analyses were run on IBM SPSS statistics V23. Results are presented as odds ratio (OR) and 95% confidence intervals (CI). For the outcome variable, “uncompleted” was considered the reference category. Therefore, all presented results have to be interpreted as the odds ratios for the completion of the next ePRO.

## Results

A total of 20.846 possible ePRO invitations could be sent to 3833 unique RA patients between 2016 upto 2020. Of these, 639 patients and 3776 possible invitations were excluded since these patients had no email address and could not receive the invitation. The definitive dataset comprised 3194 unique patients and 17.070 sent ePRO invitations. Patient characteristics are displayed in Table 1, the ePRO invitation characteristics are presented in Table 2. Of the 3194 patients, 74% (*n* = 2347) were female. A median of 6 (IQR 4–9) ePRO’s was sent to each patient and the average response rate was 40%. Of the 639 participants that had no email address, 522 were female (82%) and the average age 72, see supplementary table 1.

**Table 1:** Patient characteristics

	Unique patients: 3194	Completed ePROs: 6834 (40%)	Uncompleted ePROs: 10,236 (60%)
Age, mean (SD)	60 (14)	59 (13)	59 (14)
18–38	258 (8)	567 (38)	945 (62)
39–54	766 (24)	1787 (39)	2837 (61)
55–73	1644 (52)	3810 (44)	4949 (56)
74–99)	526 (16)	670 (31)	1505 (69)
Sex, *n* (%)			
Female	2347 (74)	5035 (40)	7726 (60)
Male	847 (26)	1799 (42)	2510 (58)
Residency, *n* (%)			
Urban	1800 (56)	4193 (43)	5489 (57)
Rural	1394 (44)	2641 (36)	4747 (64)
Disposable income per household × €1000, mean (SD)*	40* (9)	41* (9)	40* (9)
Lowest quartile (< €34,5)	365 (26)	564 (29)	1357 (71)
Second lowest quartile (€34,5-€38,5)	361 (25)	718 (36)	1256 (64)
Second highest quartile (€38,5-€45,0)	353 (25)	688 (38)	1116 (62)
Highest quartile (> €45,0)	331 (24)	697 (40)	1053 (60)
Disease duration at the time of the ePRO, years (SD)	3.5 (1.9)	3.4 (2.0)	3.6 (1.9)

* Only for patients living in Amsterdam

Numbers are *n* (%) unless otherwise stated

*SD* standard deviation, *IQR* Inter quartile range, *ePRO* electronic Patient Reported Outcome

**Table 2:** ePRO invitation characteristics

	Total sent ePROs: 17.070	Completed ePROs: 6834	Uncompleted ePROs: 10.236
Days between sent ePROs, median (IQR)	92 (58–143)	91 (57–142)	93 (59–144)
Sent ePROs per day of the week			
Monday	4107 (24)	1681 (41)	2426 (59)
Tuesday	3374 (20)	1339 (40)	2035 (60)
Wednesday	3001 (17)	1119 (37)	1882 (63)
Thursday	3402 (20)	1403 (41)	1999 (59)
Friday	3186 (19)	1292 (41)	1894 (59)
Sent ePROs per time of the day			
During working hours	14,054 (82)	5481 (39)	8573 (61)
Outside working hours	3016 (18)	1353 (45)	1663 (55)
Sent ePROs in the year after implementation			
0 (year 2016)	1879 (11)	402 (21)	1477 (79)
1	1523 (9)	574 (38)	949 (62)
2	4943 (29)	2078 (42)	2865 (58)
3	6022 (35)	2551 (42)	3471 (58)
4	2703 (16)	1229 (46)	1474 (54)

Numbers are *n* (%) unless otherwise stated

*SD* standard deviation, *IQR* Inter quartile range, *ePRO* electronic patient-reported outcome

### Predictors for uncompleted ePROs

A multivariable GEE analysis with all factors, except the disposable household income, was performed. The first step of the backward selection procedure was to exclude sex (*p* = 0.50), followed by a time of sent ePRO during working hours (*p* = 0.20), day of the week of sent ePRO (*p* = 0.14), and disease duration (*p* = 0.07). The final model contained age, urban residency, and year of sent ePRO, see Table 3. Compared to the youngest patients (the millennials), the baby-boom generation (55–73 years) had significantly higher odds of completing the ePRO (OR 1.36, 95%CI 1.15–1.60). Patients living in an urban area had lower odds for completing the following ePRO compared to patients living in urban areas (OR = 0.69, 95%CI 0.62–0.76). With advancing years after the implementation of the routine ePRO collection, the odds to report the ePRO increased significantly (year 4: OR 3.69, 95%CI 3.19–4.26).

**Table 3:** GEE result for all patients and including the factor “urban residency”

Factor	OR	95% CI		*p* value
Age				
18–38 (ref)	Ref	Ref		Ref
39–54	1.15	0.96;	1.37	0.13
55–73	1.36	1.15;	1.60	< 0.001
74–99	0.78	0.63;	0.97	0.02
Urban residency	0.69	0.62;	0.76	< 0.001
Year of sent ePRO				
2016 (ref.)	Ref	Ref		Ref
2017	2.51	2.15;	2.94	< 0.001
2018	2.94	2.57;	3.37	< 0.001
2019	2.93	2.56;	3.35	< 0.001
2020	3.69	3.19;	4.26	< 0.001

The odds ratios are corrected for the results of the previously sent ePRO and are interpreted as odds ratios for completing the next ePRO

*GEE* Generalized estimated equations, *ePRO* electronic patient-reported outcome

### Predictors for uncompleted ePROs, including disposable household income

A total of 1800 unique patients were residents of Amsterdam and were included in the analysis with disposable income as a factor. The mean age was 59 years (SD 14) and 77% was female. Of the 9682 sent invitations, 4193 (43%) were completed. In the analysis, the time of the day of the sent ePRO (*p* = 0.83) was excluded first, followed by sex (*p* = 0.72), day of the week of sent ePRO (*p* = 0.39), and disease duration (*p* = 0.03). The final model contained age, disposable household income, and year of sent ePRO, see Table 4. Compared to the youngest patients (the millennials), the baby-boom generation (55–73 years) had significantly higher odds of completing the ePRO (OR 1.39, 95% CI 1.09–1.77). Patients with higher disposable incomes had increasingly significantly higher odds for completion of the ePRO compared to patients with lower disposable income (OR 1.51, 95% CI 1.22–1.88 for the highest quartile vs. lowest quartile). With advancing years after the implementation of the routine ePRO collection, the odds to report the ePRO increased significantly (year 4: OR 3.78, 95% CI2.91–4.90).

**Table 4:** GEE result for patients residing in Amsterdam and including the factor disposable household income

Factor	OR	95% CI		*p* value
Age				
18–38 (ref)	Ref	Ref		Ref
39–54	1.22	0.95;	1.59	0.13
55–73	1.39	1.09;	1.77	0.007
74–99	0.92	0.66;	1.27	0.60
Disposable income				
1 (Lowest quartile, ref)	Ref	Ref		Ref
2	1.13	1,06;	1,63	0.01
3	1.37	1,10;	1,70	0.005
4 (Highest quartile)	1.51	1,22;	1,88	< 0.001
Year of sent ePRO				
2016 (ref.)	Ref	Ref		Ref
2017	2.71	2.02;	3.66	< 0.001
2018	2.84	2.21;	3.66	< 0.001
2019	2.96	2.31;	3.80	< 0.001
2020	3.78	2.91;	4.90	< 0.001

The odds ratios are corrected for the results of the previously sent ePRO, and are interpreted as odds ratios for completing the next ePRO

*GEE* Generalized estimated equations, *ePRO* electronic patient reported outcome

## Discussion

In this study, we investigated which patient factors, and/or timing of the prompt predicted response to ePROs sent prior to the consultation in routine clinical care in patients with RA. Patients between the age of 55–73 (baby boom generation), with higher SES, and living in a rural area had higher odds to report the ePRO. Timing of the prompt did not predict response, but the odds to report the ePRO increased with advancing years after the introduction of the pre-consultation ePRO.

The results showed that patients between the age of 55–73 had increased odds to report the ePRO, while the odds for patients older than 73 were not significantly lower. There is conflicting evidence for the association between age and eHealth usage such as reporting ePROs. Older age is often associated with lower eHealth usage, although in a published review it was concluded that there was no association between age and adherence to reporting ePROs [9, 17]. Lower eHealth usage among elderly is related to lower possession of devices such as smartphones, and lower skills that are required to use them [18]. However, the adoption of smartphones in the Netherlands among 55 + years old increased over the years to 90%, and in the UK the possession among 65 + increased from 55% in 2018 to 69% in 2021 [19]. Therefore, the main barriers to eHealth usage among the elderly might be of diminishing importance in the (Dutch) population, which could explain why we did not find a significant difference for elderly patients.

We found that patients living in a rural area had higher odds to report the ePRO compared to urban citizens. This is contradictory compared to the finding of a recent review that reported the opposite: six articles found an association between patients living in rural areas and lower eHealth usage. However, it is shown that socioeconomic status (SES) is a major confounder in this association [18, 20]. This might explain our contrasting results, as we had no information regarding the SES for our rural patients and could, therefore, not correct for SES. The analysis of urban citizens showed that a higher SES (disposable income) was associated with higher odds to report the ePRO. It is thought that patients with lower SES may have limited access to internet resources and have less developed digital health skills [20, 21]. Another reason might be that low literacy was a barrier to report the ePRO for these patients. The ability to read Dutch was a prerequisite to report the ePRO in this study, and the percentage of low literacy is considerably higher in districts in Amsterdam with lower disposable incomes (32%) compared to districts with higher disposable incomes (9%). Possible solutions to overcome this might be in-depth technical support [22] or to implement the option for spoken text. Future studies are needed to identify the exact barriers which patients with lower SES experience.

Although studies have shown that prompts play an important role in the engagement of self-monitoring programs, the timing of the sent ePRO (time of the day and day of the week) did not predict ePRO response. However, we did find that as the years after the introduction of the pre-consultation ePRO progressed, the percentage reported ePROs increased from 21% steadily to 46% after 5 years. To our knowledge, there is no comparative literature that has a follow-up duration of more than 1 year with which to compare our results, making it difficult to understand why the increase occurs. However, the increase in reported ePROs between 2019 and 2020 (+ 4%) coincides with the start of the COVID-19 pandemic. When the year 2020 is split into pre-COVID-19 months and COVID-19 months, the report rate increased from 42% in the pre-COVID months, to 48% during the COVID-19 months. It is possible that the importance of ePROs as a validated measure for disease activity increased when golden standards such as the DAS28 were difficult to collect with predominantly telephone consultations, resulting in higher report rates [23]. Unfortunately, the data was insufficient to explore if the increase remained after physical consultations became standard again.

A strength of this study is the usage of clinical care data combined with only one exclusion criteria which limited the influence of a selection bias as much as possible. This resulted in an exclusion percentage of 17% (those who had no registered email address), compared to 75% for recent trials. Still, selection bias was not negligible since the excluded patients were older and had a lower disposable income. Another strength is the long time span of the study. Studies that report clinical care data regarding the routine collection of ePROs are scarce, and trials often have a follow-up of a year at best [3, 7]. However, there are limitations to this study. First, we used retrospective data. This limited the investigated variables to the variables collected in routine care and were, therefore, unable to investigate variables such as disease activity or (e)Health literacy. Although our list of included variables does not encompass all relevant factors, it can be used to select important variables in future prospective studies. Second, we used the zip code of the patients to determine SES. And although this has proven to be a valid method, it would be more accurate when we could have determined the SES with variables on an individual level [15]. Third, bias could have occurred on the level of the rheumatologist. Discussing the results during the consultations is identified as a major facilitator to report following ePROs [24]. However, a study showed that ~ 45% of rheumatologists never or sometimes review the results of PROs, while 55% review the results often or every time [25]. Therefore, it is possible that disparities occurred in ePRO completion between sub-populations of treating rheumatologists, introducing a bias.

## Conclusion

The implementation of ePROs in daily clinical practice needs improvement since 40% of the ePROs sent prior to consultations were completed. Patients that had higher odds to report the next ePRO were between the age of 55–73, had a higher socio-economic status, and were resident in a rural area. Adoption of reporting the PRO increased over time, but the timing of the prompt did not predict response. Additional research is needed to understand ePRO completion, especially for patients with lower socio-economic status, aged below 55 and above 74, and living in urban areas.

## Supplementary Information

Below is the link to the electronic supplementary material.

Supplementary file1 (DOCX 13 KB)

## Data Availability

Data are available on request due to privacy or other restrictions.
