# Supplementary material for: Predictors for response to electronic patient-reported outcomes in routine care in patients with rheumatoid arthritis: a retrospective cohort study
Source: Rheumatol Int. 2023 Jan 30;43(4):651–7. doi: 10.1007/s00296-023-05278-6 (PMC9885920; doi:10.1007/s00296-023-05278-6)
Supplement: Supplementary file 1 — Supplementary file1 (DOCX 13 KB) [file 296_2023_5278_MOESM1_ESM.docx]

## Supplementary materials

| Supplementary table 1. Characteristics of the patients with no email address | | |
| --- | --- | --- |
| Patients | 639 |  |
| Women | 522 (82) |  |
| Age, mean (SD) | 72 (11) |  |
| 18-38 | 7 (1) |  |
| 39-54 | 40 (6) |  |
| 55-73 | 279 (44) |  |
| 74-99 | 313 (49) |  |
| Urban residency | 386 (60) |  |
| Disposable income, mean x1000€ | 35,8 |  |
| Numbers are n (%), unless otherwise stated | | |
